# Pyroptosis-related prognosis model, immunocyte infiltration characterization, and competing endogenous RNA network of glioblastoma

**DOI:** 10.1186/s12885-022-09706-x

**Published:** 2022-06-03

**Authors:** Min-Rui Ding, Yan-Jie Qu, Xiao Peng, Jin-Fang Chen, Meng-Xue Zhang, Tong Zhang, Bing Hu, Hong-Mei An

**Affiliations:** 1 Department of Neurology, Longhua Hospital, Shanghai University of Traditional Chinese Medicine, Shanghai, 200032 China; 2 Institute of Traditional Chinese Medicine in Oncology, Department of Oncology, Longhua Hospital, Shanghai University of Traditional Chinese Medicine, Shanghai, 200032 China; 3 Department of Science & Technology, Longhua Hospital, Shanghai University of Traditional Chinese Medicine, Shanghai, 200032 China

**Keywords:** Glioblastoma, Pyroptosis, Tumor microenvironment, Immunocyte infiltration, Competing endogenous RNA network

## Abstract

**Background:**

Glioblastoma (GBM) has a high incidence rate, invasive growth, and easy recurrence, and the current therapeutic effect is less than satisfying. Pyroptosis plays an important role in morbidity and progress of GBM. Meanwhile, the tumor microenvironment (TME) is involved in the progress and treatment tolerance of GBM. In the present study, we analyzed prognosis model, immunocyte infiltration characterization, and competing endogenous RNA (ceRNA) network of GBM on the basis of pyroptosis-related genes (PRGs).

**Methods:**

The transcriptome and clinical data of 155 patients with GBM and 120 normal subjects were obtained from The Cancer Genome Atlas (TCGA) and Genotype-Tissue Expression (GTEx). Lasso (Least absolute shrinkage and selection operator) Cox expression analysis was used in predicting prognostic markers, and its predictive ability was tested using a nomogram. A prognostic risk score formula was constructed, and CIBERSORT, ssGSEA algorithm, Tumor IMmune Estimation Resource (TIMER), and TISIDB database were used in evaluating the immunocyte infiltration characterization and tumor immune response of differential risk samples. A ceRNA network was constructed with Starbase, mirtarbase, and lncbase, and the mechanism of this regulatory axis was explored using Gene Set Enrichment Analysis (GSEA).

**Results:**

Five PRGs (CASP3, NLRP2, TP63, GZMB, and CASP9) were identified as the independent prognostic biomarkers of GBM. Prognostic risk score formula analysis showed that the low-risk group had obvious survival advantage compared with the high-risk group, and significant differences in immunocyte infiltration and immune related function score were found. In addition, a ceRNA network of messenger RNA (CASP3, TP63)–microRNA (hsa-miR-519c-5p)–long noncoding RNA (GABPB1-AS1) was established. GSEA analysis showed that the regulatory axis played a considerable role in the extracellular matrix (ECM) and immune inflammatory response.

**Conclusions:**

Pyroptosis and TME-related independent prognostic markers were screened in this study, and a prognosis risk score formula was established for the first time according to the prognosis PRGs. TME immunocyte infiltration characterization and immune response were assessed using ssGSEA, CIBERSORT algorithm, TIMER, and TISIDB database. Besides a ceRNA network was built up. This study not only laid foundations for further exploring pyroptosis and TME in improving prognosis of GBM, but also provided a new idea for more effective guidance on clinical immunotherapy to patients and developing new immunotherapeutic drugs.

**Supplementary Information:**

The online version contains supplementary material available at 10.1186/s12885-022-09706-x.

## Background

Glioblastoma (GBM) is the most common malignant tumor of the central nervous system [1]. It is characterized by invasive growth and easy recurrence [2]. Recently, despite continuous developments in treatment methods, such as surgery, radiotherapy, and chemotherapy have been reported, the median survival time of patients remains 12–15 months, and no substantial improvement has been achieved yet [3]. Some reasons are related to the potential molecular and cell heterogeneity of GBM [4]. Therefore, exploring the potential molecular mechanism of GBM and determining the key therapy targets are of important significance.

Pyroptosis is an inflammatory form of programmed cell death, and its characteristic lies in membrane perforation, cell swelling, plasma membrane rupture, and intracellular content release [5]. Pyroptosis is mainly mediated by the gasdermin family, which includes GSDMA, GSDMB, GSDMC, GSDMD, GSDME/DFNA5, and DFNB59. Except DFNB59, the other members of the gasdermin family are activated by cleaving the C-terminal and N-terminal domains. Specifically, the N-terminal segments form holes in the plasma membrane, thus resulting in the swelling and rupture of cells [6]. Pyroptosis cells release proinflammatory molecules to the extracellular environment, thus triggering inflammation and immunoreaction [7]. Pyroptosis induced by the combination of anti-tumor drugs and autophagy inhibitors can inhibit GBM growth and increase survival rate [8]. Ren et al. [9] reported that activating NF-κB/NLRP3/GSDMD pathway can trigger pyroptosis of GBM and thereby inhibit GBM growth. Pyroptosis plays a crucial role in the pathogenesis and progress of various malignant tumors, including GBM [10].

The Tumor microenvironment (TME) is the site of tumor cell growth and development. It is composed of stromal cells, signaling molecules, immune cells and extracellular matrix (ECM) [11]. TME plays an important role in the progression and treatment resistance of GBM [12]. The GBM microenvironment contains a large number of innate immune cells (monocytes, macrophages, mast cells, microglia, and neutrophils), T cells, vascular cells, astrocytes, and oligodendrocytes [13]. It has complex and dynamic communication modes with tumor cells, which is essential for tumor proliferation, migration and immunosuppression [14]. Interactions between tumor cells and infiltrating immunocytes are generally regulated by competing endogenous RNA (ceRNA) networks, which are composed of messenger RNAs (mRNAs), long noncoding RNAs (lncRNAs), and microRNAs (miRNAs). These networks can regulate the post-transcription of oncogenes and tumor suppressor genes and the interactions between protein and genes, thus regulating the biological behavior of tumors, particularly invasion and metastasis [15]. Therefore, comprehensive understanding of TME cell infiltration characteristics related to multiple PRGs and ceRNA networks might provide a new perspective for the study of the potential mechanisms of the occurrence and development of GBM and prediction of its responses to immunotherapeutic approaches.

**Fig. 1:**
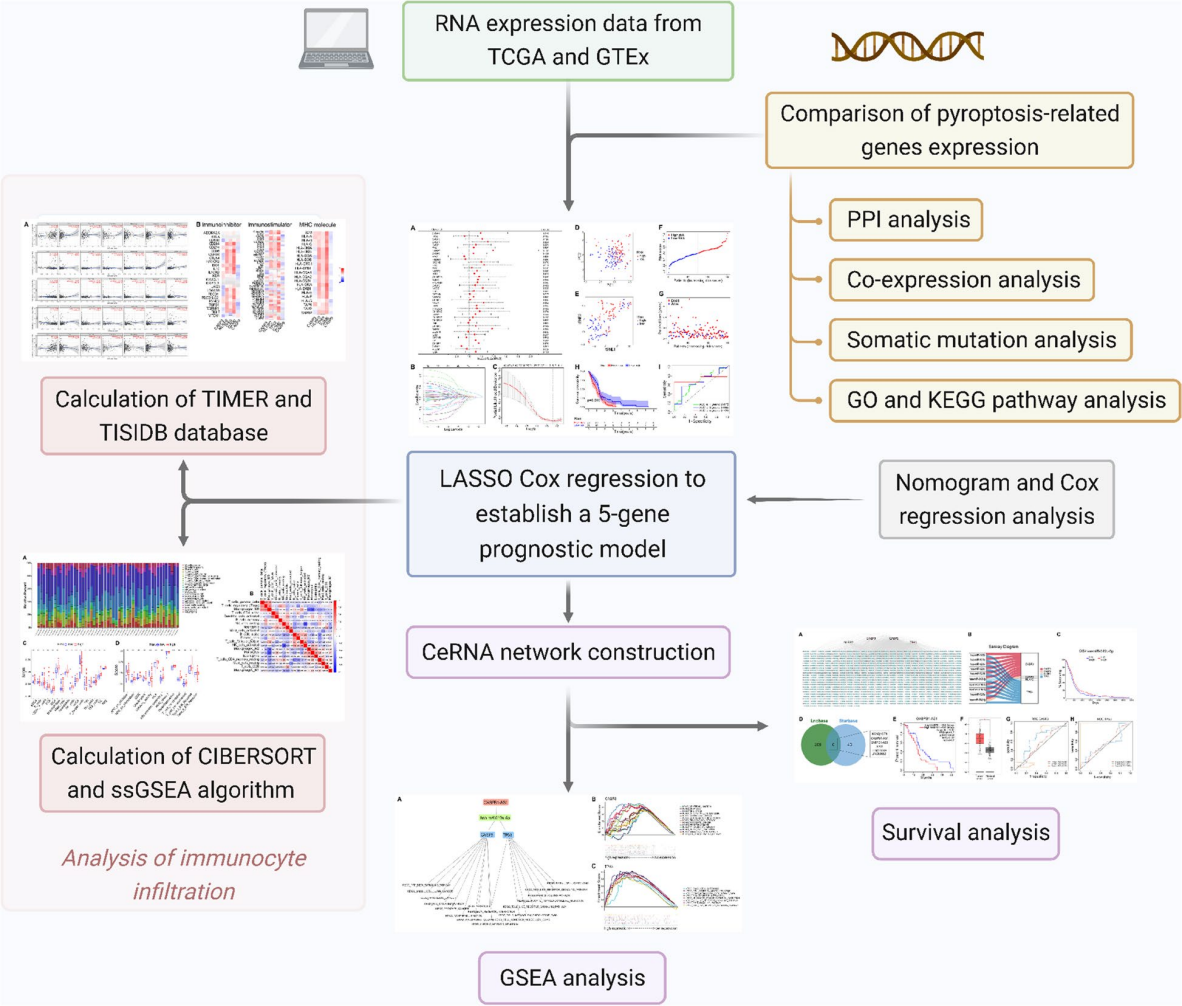
Workflow diagram. The specific workflow graph of this work

In the present study, potential independent prognosis pyroptosis-related genes (prognosis PRGs) of GBM were screened comprehensively, and a prognostic model was constructed. Independent PRGs and the immunoreactions of tumors are associated. Subsequently, a prognosis risk evaluation formula was established on the basis of independent prognosis PRGs, and the TME cell infiltration characterization of differential risk scoring of GBM was assessed. The results demonstrated that independent prognosis PRGs played a considerable role in the formation of the TME characterization features of GBM. These genes might be attributed to the potential immunotherapeutic targets and biomarkers of GBM. The ceRNA network of mRNA (CASP3, TP63)–microRNA (hsa-miR-519c-5p)–lncRNA (GABPB1-AS1) was constructed for the first time on the basis of a pyroptosis-related prognostic model with bioinformatics tools for the reverse prediction of target genes. The possible action mechanisms of this regulation axis in the growth, invasion, and metastasis of GBM were identified using Gene Set Enrichment Analysis (GSEA) for the interpretation of the prognosis value of PRGs in GBM and relevant molecular mechanisms. The workflow is shown in Fig. 1.

## Materials and methods

### Datasets and preprocessing

The RNA-sequencing (RNA seq) data of 155 GBM and five normal subjects and relevant clinical data were collected from The Cancer Genome Atlas (TCGA) database [16] (https://portal.gdc.cancer.gov/). The RNA seq data of the brain tissues of 115 normal subjects were obtained from the Genotype-Tissue Expression (GTEx) project [17] (https://www.gtexportal.org/). Single-nucleotide polymorphism (SNP) data were downloaded from TCGA. The unit of RNA seq data was normalized as FPKM.

### Identification of differentially expressed PRGs

A total of 49 PRGs were extracted from GSEA-MSigDB (http://www.gsea-msigdb.org/gsea/login.jsp), Reactome (https://reactome.org/), Harmonizome (https://maayanlab.cloud/Harmonizome/), and Pubmed (https://pubmed.ncbi.nlm.nih.gov/). By using R software (version 4.1.1) and “ggpubr” R package, 42 PRGs were screened according to *P* < 0.05. The protein–protein interaction (PPI) networks of the PRGs were presented using the Search Tool for the Retrieval of Interacting Genes (STRING; version 11.5, https://string-db.org/) and Cytoscape software (version 3.8.2). The “igraph” and “reshape2” R packages were used in building a differential gene co-expression network.

### Analysis of the somatic mutation characteristics of PRGs

The somatic mutation frequency of the 49 PRGs was analyzed and visualized with the “maftools” R package. A mutation abstract plot and oncoplot were then generated.

### Functional enrichment analysis

We performed Gene Ontology (GO) [18] (http://geneontology.org/) enrichment and Kyoto Encyclopedia of Gene and Genomes (KEGG) [19] (www.kegg.jp/kegg/kegg1.html) pathway analyses with the “clusterProfiler” R package. The biological processes, cell components, molecular functions, and KEGG pathways of PRGs were visualized using the “ggplot2” and “GOplot” R packages.

### Establishment and validation of the pyroptosis-related gene prognostic model

Cox regression analysis was used in evaluating the prognosis value of the PRGs. Forest map was used to display the hazard ratio (HR), 95% confidence interval (CI), and *P* values of each variable. HR was used in determining the protective genes (HR < 1) and risk genes (HR > 1). Based on these PRGs related with prognosis, a Lasso (Least absolute shrinkage and selection operator) Cox regression model was constructed with the “glmnet” R package to screen optimal prognosis PRGs. The optimal value of penalty parameter (λ) was selected according to 1000 cross-validation runs. The weighted regression coefficient and expression levels of the prognosis PRGs were extracted, and the risk score was calculated using the following formula as the index for measuring the survival risk of each patient: ${\sum}_{\mathrm{i}}^5{\upbeta}_{\mathrm{i}}\times {\mathrm{X}}_{\mathrm{i}}$ (β: coeffificients, X: gene expression level). According to the median risk score, patients with GBM in the TCGA were divided into low-risk (risk score < median risk value) and high-risk (risk score > median risk value) groups. The data visualization algorithms of principal component analysis (PCA) and t-distributed stochastic neighbor embedding (t-SNE) were performed using the “Rtsne” and “ggplot2” R packages. The survival times of the two groups were compared through Kaplan-Meier analysis. Receiver operating characteristic (ROC) analysis was carried out with the “survival,” “survminer,” and “timeROC” R packages for the evaluation of the prediction accuracy of different genes and risk scores.

### Independent prognostic analysis and clinical value of the risk model

To determine whether the risk score from the gene characteristic model can be used as an independent prognosis factor for patients with GBM, we conducted a univariate and multivariate Cox regression analysis to the five prognosis PRGs. A clinical feature heatmap was built by extracting the clinical information (age and gender) of patients in the TCGA group and combining with risk score in the regression model. A nomogram can be used in predicting the prognosis of cancer. In this study, a prognostic nomogram was built by including the prognosis PRGs and used in analyzing the probability of 1-, 2-, and 3-year survival times of patients with GBM. This model was implemented using the “rms” R package. The calibration curve of the nomogram was plotted for the evaluation of its prediction accuracy.

### Immunocyte infiltration analysis

The correlation between prognosis PRGs and immunocyte levels (e.g., B cell, CD8+ T cell, CD4+ T cell, macrophage, neutrophil, and dendritic cell) was detected with Tumor IMmune Estimation Resource (TIMER) database (http://timer.cistrome.org/). The correlation between the expression of prognosis PRGs and immunoregulators (including immunoinhibitor, immunostimulator and MHC molecule) was analyzed using the TISIDB database (http://cis.hku.hk/TISIDB/).

To quantify the relative proportions of immunocytes in the high-risk and low-risk groups, we calculated immunocyte infiltration with the CIBERSORT algorithm. Samples were screened according to the standard (*P* < 0.05), and the percentage of each immunocyte type in the samples was calculated, which were exhibited in a bar diagram. Correlation heatmap analysis was carried out with the “corrplot” R package, which disclosed the correlations among 22 types of immunocytes. Additionally, the scores of 16 immunocytes and 13 immune-related functions were calculated with the ssGSEA algorithm for the analysis of the differential expression levels in the high-risk and low-risk groups.

### CeRNA network construction

To elaborate the potential functions of prognosis PRGs in GBM, a ceRNA network was built. Starbase (version 2.0, http://starbase.sysu.edu.cn/) and MirTarbase (version 9.0 beta, http://mirtarbase.cuhk.edu.cn/) were used in predicting the target miRNA of the prognosis PRGs. The top 10 highly connected miRNAs were screened by calculating the degree among various nodes with the CytoHubba plug-in of Cytoscape software (version 3.8.2), and the prognosis values of the screened miRNAs were analyzed using the Oncolnc database (http://www.oncolnc.org/). Based on above miRNA, Lncbase (version v.2, https://carolina.imis.athena-innovation.gr/diana_tools/web/index.php?r=lncbasev2/) and Starbase were used in predicting the lncRNA targets that interacted with miRNAs, and the intersection of the lncRNA targets were screened with a Venn diagram (https://bioinfogp.cnb.csic.es/tools/venny/). The expression and prognosis conditions of the lncRNA targets were analyzed with GEPIA2 (http://gepia2.cancer-pku.cn/).

### Gene set enrichment analysis

To explore the influences of gene expression on this pathway, GSEA was carried out. The first 50% of PRG expression was set as the high-expression group, and the rest was set as the low-expression group. GSEA was carried out with GSEA software (version 4.1.0).

## Results

### Defining the expression of PRGs in GBM

**Fig. 2:**
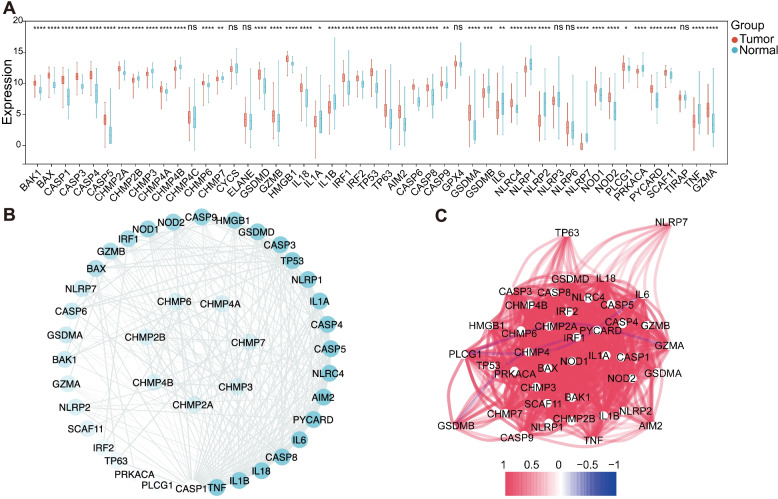
Expression and interactions of the PRGs. **A** The expression of 49 PRGs in GBM and normal brain tissues. The upper and lower ends of the boxes represented the interquartile range of values. The lines in the boxes represented the median value. **B** The PPI network showing the interactions of differentially expressed PRGs (minimum required interaction score = 0.4). The color depth of the node represented the degree. **C** The correlation network of differentially expressed PRGs (red line: positive correlation, blue line: negative correlation. The depth of color reflected the intensity of correlation)

A total of 42 differential expressed PRGs in GBM were recognized through the comparison of the differential expressions of 49 PRGs in 120 normal subjects and 155 GBM from the TCGA and GTEx database (*P* < 0.05). The expression levels of 13 genes (NLRP2, NLRP7, TNF, IL1B, IL6, NLRP1, IL1A, PRKACA, GSDMB, CHMP3, CHMP4B, CHMP2B, and CHMP7) in GBM were downregulated relative to those in the brain tissues of normal subjects, whereas the expression levels of 29 genes (PLCG1, CASP9, CHMP6, SCAF11, CHMP4A, CHMP2A, HMGB1, IRF2, NLRC4, GZMB, NOD1, TP63, BAK1, CASP8, BAX, IRF1, CASP3, PYCARD, IL18, GSDMD, AIM2, GSDMA, NOD2, GZMA, CASP5, CASP6, TP53, CASP1, and CASP4) were upregulated (Fig. 2A). To further explore the interactions of these differentially expressed PRGs, we carried out PPI analysis. As shown in Fig. 2B, the minimum required interaction score was set at 0.4, and the degree of each node in the network was expressed and arranged according to color. The top 10 genes in the term of degree were TNF, IL1B, IL18, CASP8, IL6, PYCARD, AIM2, NLRC4, AIM2, and NLRC4. The correlation network results of the differentially expressed PRGs are shown in Fig. 2C. Most differentially expressed PRGs were positively related, whereas negative correlations between GZMA and CHMP2A, between IL6 and PLCG1, and between GSDMB and CHMP4A were found.

### Analysis of the somatic mutation characteristics of PRGs

**Fig. 3:**
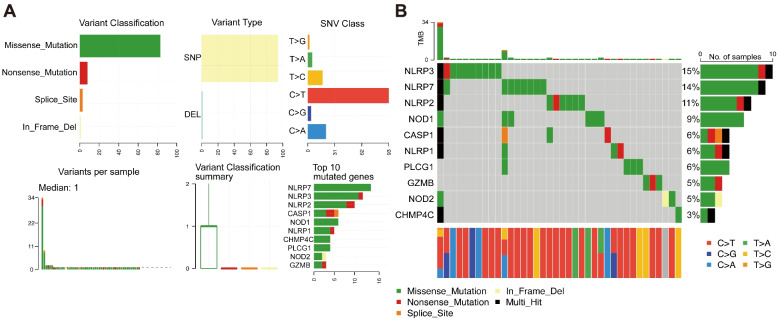
Somatic mutations and mutational signatures of PRGs in GBM. **A** The summary plot of somatic mutations of PRGs in GBM. **B** The oncoplot of mutation frequency of PRGs in GBM

Somatic mutation frequency of 49 PRGs in GBM was analyzed. The results demonstrated that missense mutation was the most common mutation classification, and SNP was the most common variant type. Single-nucleotide variation mainly occurred in the form of C > T (Fig. 3A). In the GBM samples, the top 10 mutation genes related to pyroptosis were NLRP3, NLRP7, NLRP2, NOD1, CASP1, NLRP1, PLCG1, GZMB, NOD2, and CHMP4C (Fig. 3B).

### Functional enrichment analysis of the PRGs

**Fig. 4:**
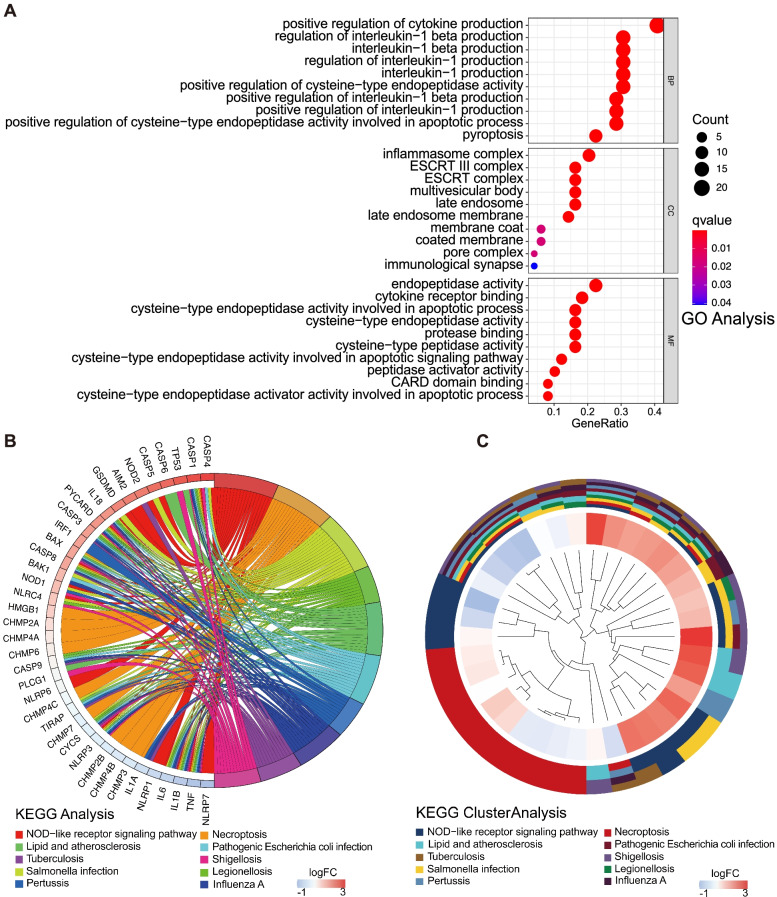
Functional enrichment analysis of PRGs in GBM. **A** GO enrichment analyses of PRGs. **B** KEGG pathway enrichment analyses of PRGs. **C** Cluster plot describing the top 10 results of the KEGG enrichment analysis. Cluster plot displaying a circular dendrogram of the clustering of the expression profiles. The inner ring shows the color-coded logFC, the outer ring the assigned functional terms

GO and KEGG databases were used in analyzing the related pathways of differentially expressed PRGs and clarifying their functions. As shown in Fig. 4A, the differentially expressed PRGs were mainly enriched in the positive regulation of cytokine production, regulation of interleukin-1 production, positive regulation of cysteine-type endopeptidase activity, and pyroptosis biological processes. In addition, the genes were mainly related to multiple cell components, such as inflammasome complex, ESCRT complex, multivesicular body, late endosome membrane, pore complex, and immunological synapse. Moreover, molecular function analysis verified that the PRGs were correlated to endopeptidase activity, cytokine receptor binding, protease binding, cysteine-type peptidase activity, peptidase activator activity, and CARD domain binding. KEGG pathway enrichment analysis demonstrated that the PRGs were closely related to NOD-like receptor signaling pathway, necroptosis, *Salmonella* infection, legionellosis, lipid and atherosclerosis, and pathogenic *Escherichia coli* infection (Fig. 4B and C).

### Construction of the prognostic PRG model

**Fig. 5:**
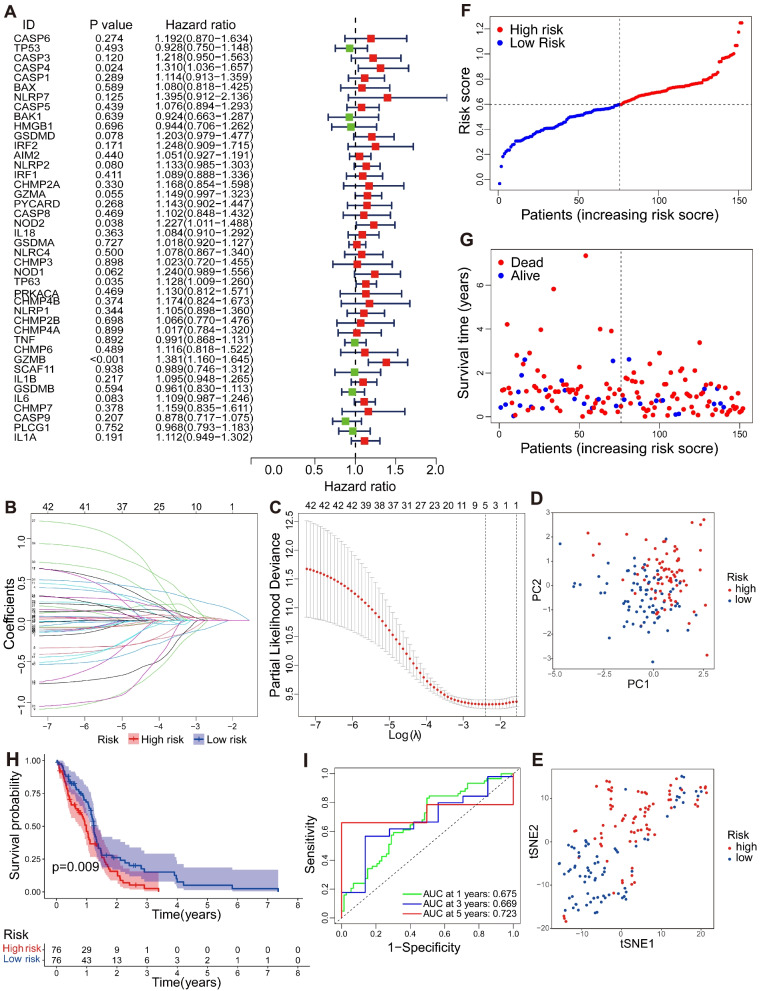
Construction of the prognostic PRG model. **A** Univariate Cox regression analysis of GBM for each prognostic PRG. **B** Distribution of Lasso coefficient for prognostic PRGs. **C** Partial likelihood deviance of the Lasso coefficient distribution. **D** PCA plot of GBM patients based on the risk score. **E** t-SNE plot of GBM patients based on the risk score. **F** Distribution of GBM patients based on the risk score. **G** Distribution of GBM patients based on survival status. **H** Overall survival curves for GBM patients in high−/low-risk group. **I** ROC curves of measuring the predictive value

According to the survival-related genes screened through univariate Cox regression analysis, 34 differentially expressed PRGs (CHMP4A, GSDMA, CHMP3, AIM2, CHMP2B, CASP5, NLRC4, BAX, IL18, IRF1, IL1B, CASP8, NLRP1, IL6, IL1A, CASP1, CHMP6, TP63, PRKACA, NLRP2, PYCARD, GZMA, CHMP7, CHMP2A, CHMP4B, CASP6, GSDMD, CASP3, NOD2, NOD1, IRF2, CASP4, GZMB, and NLRP7) had HRs of > 1, indicating that they were related to increased risk. The other genes (CASP9, BAK1, BAK1, HMGB1, GSDMB, PLCG1, SCAF11, and TNF) had HRs of < 1 and were protective factors (Fig. 5A).

To evaluate the prognosis value of these differentially expressed PRGs, a Lasso-Cox regression model was built with “glmnet” R package and used in further screening. According to the minimum penalty parameter (λ), five of the 42 differentially expressed PRGs were retained, namely, CASP3, NLRP2, TP63, GZMB, and CASP9 (Fig. 5B and C). A prognosis risk score formula was established according to weighted regression coefficient and expression levels based on multivariate Cox regression analysis: Risk score = 0.0046 × (expression value of CASP3) + 0.0060 × (expression value of NLRP2) + (0.0227) × (expression value of TP63) + 0.1804 × (expression value of GZMB) - (0.0481) × (expression value of CASP9). According to the median score calculated in the prognosis risk score formula, 152 patients with GBM were divided into low- and high-risk groups. PCA and t-SNE analysis demonstrated that patients with different risks can be divided into two types (Fig. 5D and E). The risk score distribution and survival states of the patients are shown in Fig. 5F and G. As risk score increased, the risk of death in the patients increased, whereas survival time was shortened. According to the Kaplan–Meier survival curve, the low-risk group had more obvious survival advantages (*P* = 0.009, Fig. 5H). The area under curve values of the 1-, 3-, and 5-year ROC curves were 0.675, 0.669, and 0.723, respectively (Fig. 5I), indicating that the pyroptosis model can predict the 1-, 3-, and 5-year survival rates of patients with GBM.

### Independent prognostic analysis and nomogram construction based on risk model

**Fig. 6:**
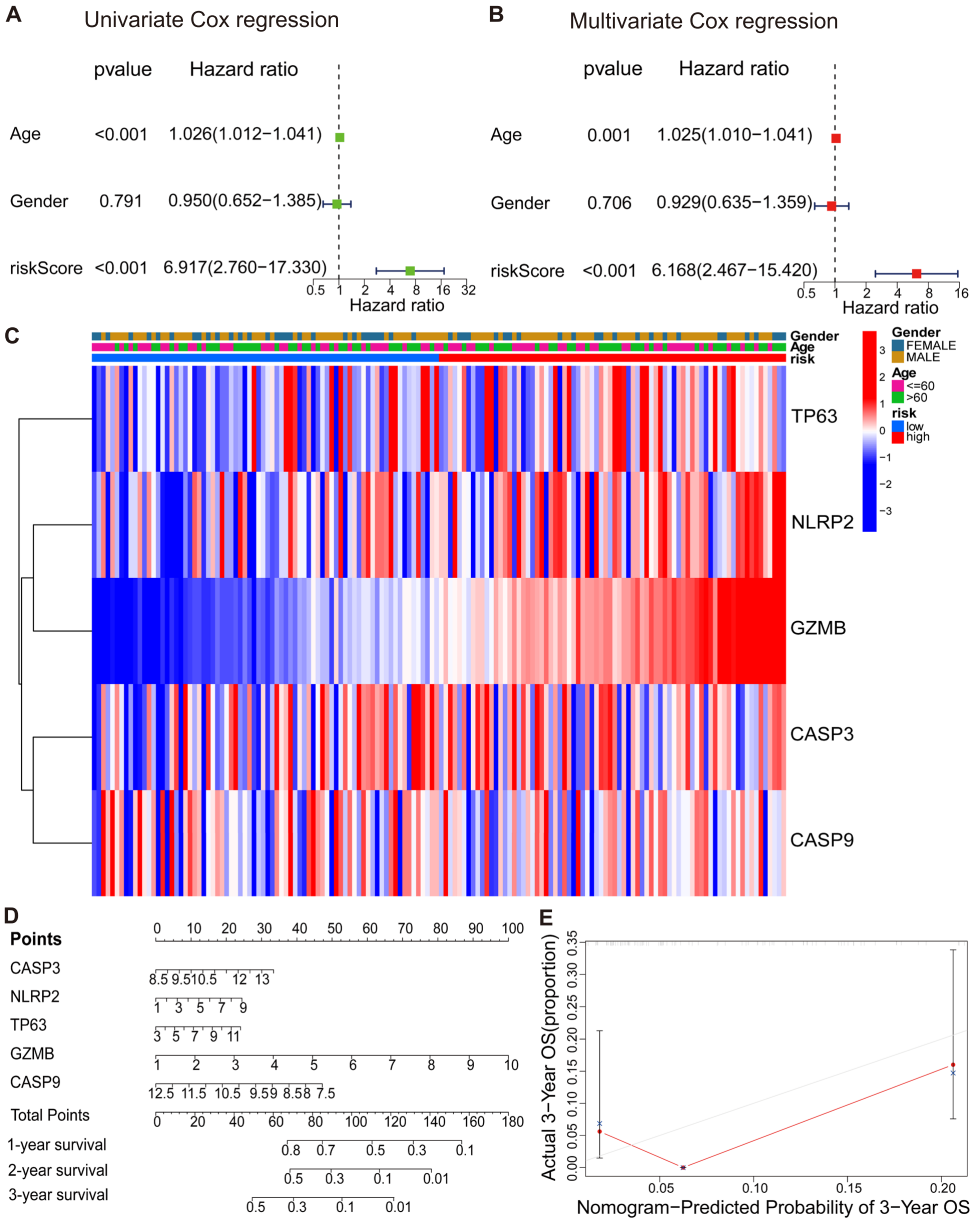
Construction of univariate and multivariate Cox regression analysis of risk score and nomogram of prognostic PRGs. **A** The *P* value and hazard ratio of the components involved in univariate Cox regression related to clinical parameters and risk score of GBM. **B** The *P* value and hazard ratio of the components involved in multivariate Cox regression related to clinical parameters and risk score of GBM. **C** Heatmap for the connections between clinical characteristics and high−/low-risk groups. **D** The nomogram for predicting 1-, 2-, and 3-year overall survival rate in GBM patients. **E** Calibration curve of the overall survival nomogram model

Whether risk score from the gene characteristic model can be used as an independent prognosis biomarker of GBM was determined using univariate and multivariate Cox regression analysis. Univariate and multivariate Cox regression analyses demonstrated that the risk score was an independent factor that influenced the prognosis of patients with GBM (HR = 6.917, 95% CI: 2.760–17.330 and HR = 6.168, 95% CI: 2.467–15.420; Fig. 6A and B). In addition, the clinical feature heatmap in Fig. 6C showed that the gender and age of patients showed no significant differences between the low- and high-risk groups (*P* > 0.05).

To discuss the importance of these five prognosis PRGs to the prognosis prediction of patients with GBM, we constructed a nomogram to visualize the Cox regression model as the survival probability of patients. The performance of the model was evaluated using a calibration curve. Compared with the ideal model, the predicted total 3-year survival rate of patients with GBM based on prognosis PRGs was slightly different from the practical value. All the five prognosis PRGs had good prognosis prediction ability for GBM (Fig. 6D and E).

The risk score was closely related to the expression levels of the five prognosis PRGs. It was an important factor for predicting the prognosis results of patients with GBM and had good prediction ability. The five prognosis PRGs might participate in the progress of GBM.

### Correlation between the expression of prognostic PRGs and immunocyte levels and immunoregulators in GBM

**Fig. 7:**
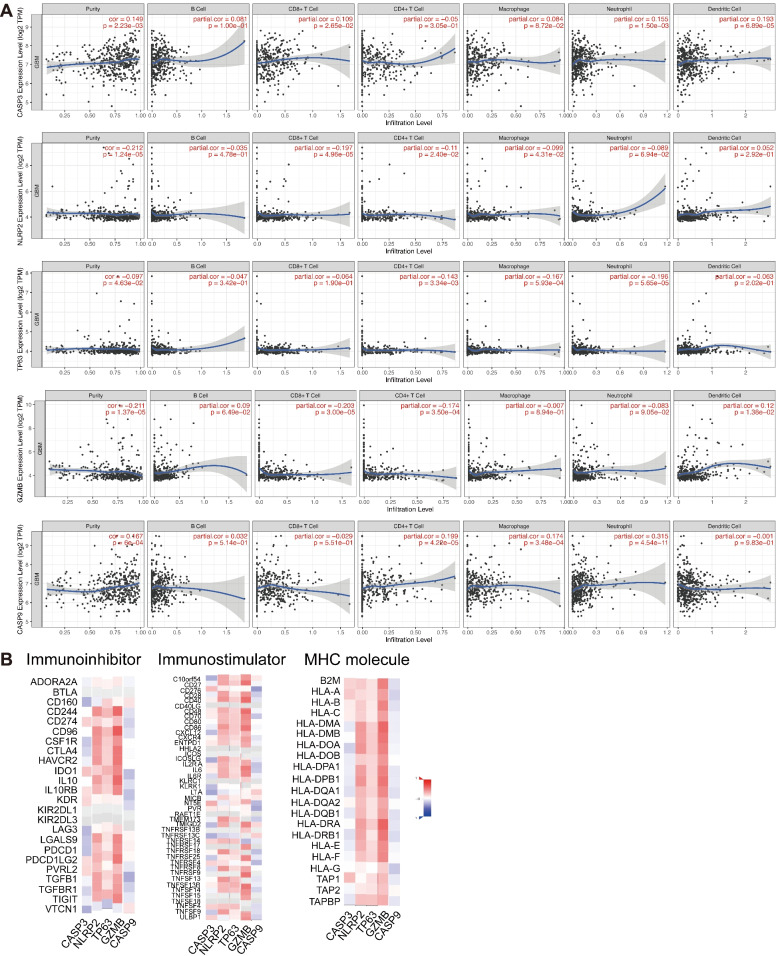
Association between five prognostic PRGs and tumor immune infiltration as well as immunoregulators in GBM. **A** The association between the abundance of immune cells and the expression of CASP3, NLRP2, TP63, GZMB and CASP9 in GBM. **B** The correlations between the expression of CASP3, NLRP2, TP63, GZMB, and CASP9 and immunoinhibitors, immunostimulators and MHC molecules were calculated using the TISIDB database

Using the TIMER database, we detected the correlation of five prognosis PRGs with the levels of immunocyte infiltration in GBM (Table S1). As shown in Fig. 7A, CASP3 was significantly associated with CD8+ T cell (*r* = 0.109; *P* = 2.65e-02), macrophage (*r* = 0.084; *P* = 8.72e-02), neutrophil (*r* = 0.155; *P* = 1.50e-03), and dendritic cell (*r* = 0.193; *P* = 6.89e-05). NLRP2 was significantly associated with CD8+ T cell (*r* = − 0.197; *P* = 4.96e-05). TP63 was significantly associated with CD4+ T cell (*r* = − 0.143; *P* = 3.34e-03), macrophage (*r* = − 0.167; *P* = 5.93e-04), and neutrophil (*r* = − 0.196; *P* = 5.65e-05). GZMB was significantly associated with CD8+ T cell (*r* = − 0.203; *P* = 3.00e05) and CD4+ T cell (*r* = − 0.174; *P* = 350e-04). CASP9 was significantly associated with CD4+ T cell (*r* = 0.199; *P* = 4.22e-05) and macrophage (*r* = 0.174; *P* = 3.48e-04). The above results showed a significant correlation between prognosis PRGs and tumor immunocyte infiltration.

To further address the influences of prognostic PRGs on tumor immune response, the correlation between the expression of prognostic PRGs and immunoregulators was calculated according to the TISIDB database. CASP3 and CASP9 were negatively correlated with immunoinhibitor, immunostimulator and MHC molecule in GBM, whereas NLRP2, TP63, and GZMB were positively related to them (Fig. 7B). Hence, the expression levels of the prognosis PRGs were closely related to the abundance of immunocyte infiltration and immunoregulators.

### Immunocyte infiltration between the high-risk and low-risk groups

**Fig. 8:**
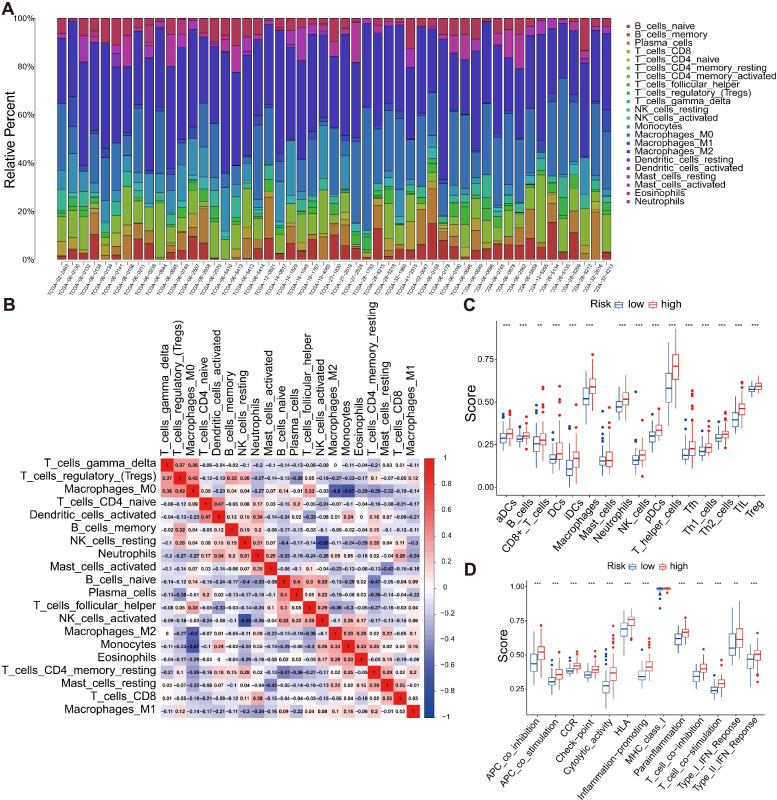
Landscape of immunocyte infiltration. **A** The relative percentage of 22 types of immune cells. **B** Correlation matrices for all 22 immune cell subtype components. Both horizontal and vertical axes showed immune cell subtypes. The higher, lower, and the same correlation levels of immune cell subtype components were shown in red, blue, and white, respectively. **C** Boxplots of the score differences of 16 immune cells between high-risk and low-risk groups. **D** Boxplots of the score differences of 13 immune-related functions between high-risk and low-risk groups

We used the CIBERSORT algorithm to analyze the abundance of immunocyte infiltration between the high-risk and low-risk groups in patients with GBM. The high-risk group presented higher distribution levels of monocytes, activated dendritic cells, activated mast cells, and eosinophils and lower distribution levels of resting NK cells and resting mast cells (Fig. 8A). According to the correlations among 22 types of immunocytes, activated dendritic cells were positively related with naive CD4 T cells (*r* = 0.47), whereas M0 macrophages were negatively related with regulatory T cells (*r* = 0.42). The resting NK cells were negatively correlated with activated NK cells (*r* = − 0.69), monocytes, and M2 macrophages were negatively correlated with M0 macrophages (*r* = − 0.67; *r* = − 0.6; Fig. 8B).

The differential expression of immunocyte infiltration and immune-related function score between the high- and low-risk groups was analyzed by ssGSEA algorithm. Significant differences in B cells, CD8+ T cells, dendritic cells, macrophages, neutrophils, NK cells, T helper cells, tumor-infiltrating lymphocytes, and regulatory T cells were observed between the groups (Fig. 8C). Moreover, significant differences in the scores of APC co-inhibition, APC co-stimulation, CCR, check point, cytolytic activity, HLA, inflammation-promoting process, parainflammation, T-cell co inhibition, T-cell co stimulation, type I FN response, and type II FN response were found between the groups (Fig. 8D).

Significant difference in immunocyte infiltration was found between the groups. Therefore, monocytes, dendritic cells, eosinophils, NK cells, and mast cells might be potential immunocytes that play important roles and participated in the progress of GBM.

### Construction of the network of mRNA–miRNA–lncRNA

**Fig. 9:**
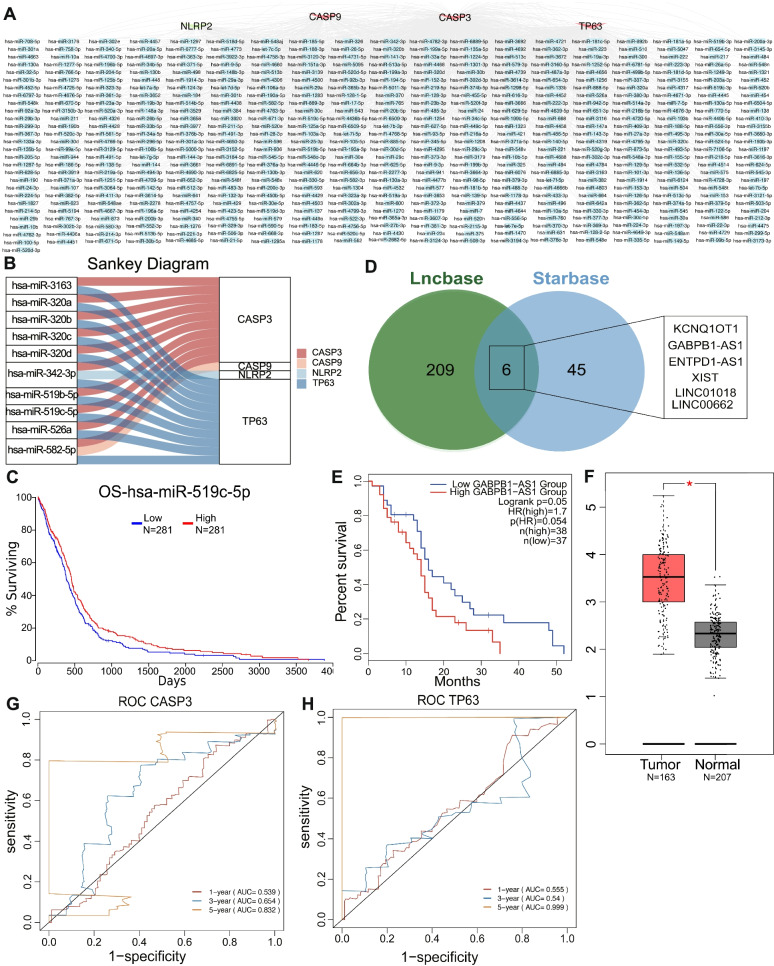
Construction of the ceRNA network. **A** The result of miRNA targets of prognosis PRGs were predicted by mirTarBase and Starbase databases. Red nodes represented highly expressed mRNA, green node represented mRNA with low expression, and blue nodes represented miRNA targets. **B** The relationship between mRNAs and their corresponding highly connected miRNAs. **C** The survival analysis results of miRNA hsa-miR-519c-5p in GBM. **D** The results of lncRNA targets predicted by Lncbase and Starbase databases. **E** The survival analysis of lncRNA GABPB1-AS1 in GBM. **F** The differential expression of lncRNA GABPB1-AS1 in GBM and normal samples. **G** ROC of HUB gene CASP3 in the ceRNA network. **H** ROC of HUB gene TP63 in the ceRNA network

To elaborate the potential molecular mechanism of the five pyroptosis-related mRNAs (CASP3, NLRP2, TP63, GZMB, and CASP9) which had prognosis values in GBM, an mRNA–miRNA–lncRNA co-expression network was built. miRNA targets that combined with these five prognostic PRGs were predicted reversely by using mirTarBase and Starbase. A total of 514 miRNA targets of CASP3, NLRP2, TP63, and CASP9 were obtained (Fig. 9A). The degree values among miRNAs nodes were calculated using the CytoHubba plug-in of Cytoscape software, and the top 10 highly connected miRNAs were determined (hsa-miR-342-3p, hsa-miR-582-5p, hsa-miR-3163, hsa-miR-320a, hsa-miR-320c, hsa-miR-320d, hsa-miR-320b, hsa-miR-526a, hsa-miR-519b-5p, and hsa-miR-519c-5p; Fig. 9B). The OncoLnc database determined a miRNA, hsa-miR-519c-5p, and GBM patients with high expression levels of hsa-miR-519c-5p. These patients had relatively high total survival rates (*P* = 0.0337; Fig. 9C). Based on the above miRNA, the upstream lncRNA targets were explored for the construction of a miRNA–lncRNA axis. As shown in Fig. 9D, for the intersection lncRNAs of Lncbase and Starbase, KCNQ1OT1, GABPB1-AS1, ENTPD1-AS1, XIST, LINC01018, and LINC00662 were determined as targets. According to survival analysis and differential expression of the lncRNA targets, only GABPB1-AS1 significantly decreased the survival probability of patients with GBM (*P* = 0.05; Fig. 9E) and it was upregulated significantly in the GBM samples (|log2FC| < 1; *P* < 0.01; Fig. 9F). These results indicated that the regulatory axis of lncRNA GABPB1-AS1/hsa-miR-519c-5p/CASP3/TP63 plays an important role in GBM. We further applied ROC curves to analyze the predictive ability of CASP3 and TP63 genes for disease. The results showed that the AUC values of CASP3 gene were 0.539, 0.654, and 0.832 at 1-, 3-, and 5-year (Fig. 9G), respectively. The AUC values for the TP63 gene at 1-, 3-, and 5-year were 0.555, 0.540, and 0.999 (Fig. 9H), respectively. This demonstrated that the HUB genes CASP3 and TP63 can predict the 1-, 3-, and 5-year survival rates of GBM patients.

### GSEA enrichment

**Fig. 10:**
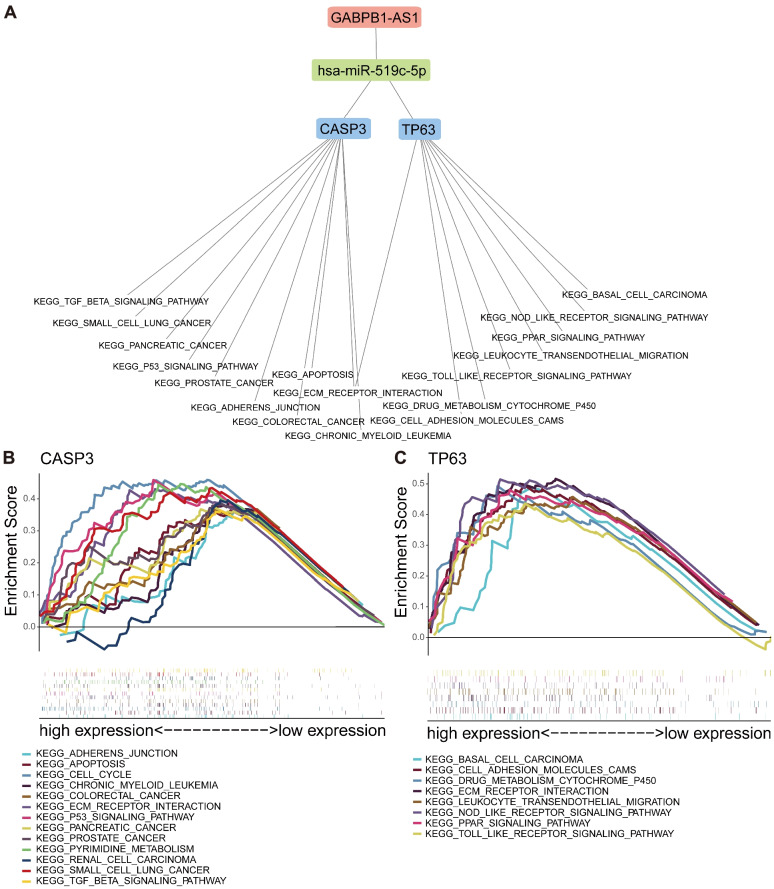
Single-gene GSEA enrichment results of two mRNAs. **A** The results of using mRNA in the stepwise reverse prediction of miRNA and LncRNA and construction of a ceRNA network. **B** Single gene enrichment analysis of CASP3. **C** Single gene enrichment analysis of TP63

The GSEA results showed that CASP3 and TP63 were enriched on ECM-related signaling pathways (e.g. ECM receptor interaction, adherens junction, and TGFβ signaling pathways) and immune-related signaling pathways (e.g. Toll-like receptor and NOD-like receptor signaling pathways; Fig. 10). This result showed that the two pyroptosis-related mRNAs are closely related to the growth, metastasis, and diffusion of GBM, and they play important roles in the immunoregulation of GBM TME.

## Discussion

Pyroptosis is closely related to malignant tumor [10], and TME plays an important role in the progress, metastasis, and treatment resistance of GBM [12]. Most studies only focused on a single PRG or TME cell type, and antitumor effect develops through interactions among tumor suppressor factors and system coordination [20]. Therefore, the effects of several PRGs on immunocyte infiltration characterization in TME were discussed by combining the ceRNA network, which is useful in elucidating the potential mechanism of initiation and development of GBM and prediction of the responses of immunotherapeutic approaches. Hence it can guide immunotherapy more effectively in clinics.

This study elaborated the differential expression of 49 PRGs in GBM samples and normal tissues and disclosed potential signaling pathways. The expression levels of NLRP2, NLRP7, TNF, IL1B, IL6, and NLRP1 in GBM were downregulated relative to those in normal tissues, whereas the expression levels of PLCG1, CASP9, CHMP6, SCAF11, and CHMP4A were upregulated. According to GSEA results, the differential genes mainly participated in pyroptosis, cytokine production, NOD-like receptor signaling pathway, necroptosis, and other signaling pathways.

Subsequently, a prediction gene model based on five prognosis PRGs (CASP3, NLRP2, TP63, GZMB, and CASP9) was constructed by using Lasso-Cox regression analysis. The results demonstrated that the five prognosis PRGs had good prognosis prediction ability for GBM. According to the TIMER and TISIDB database, the correlations between prognosis PRGs and abundance of immunocyte infiltration in GBM were evaluated. The expression levels of the five prognosis PRGs were closely related to CD8+ T cells, neutrophils, macrophages, dendritic cells, and CD4+ T cells, as well as immunoregulators. In addition, a prognosis risk score formula was established using the pyroptosis-related prognostic model. Patients with GBM were divided into low-risk and high-risk groups. The Kaplan-Meier survival curve showed that the low-risk group had obvious survival advantages. Notably, the high-risk and low-risk groups showed significant differences in immunocyte infiltration. These differences in immunocyte infiltration between the high-risk and low-risk groups has been found to exist in a variety of tumors [21–24]. Our results showed that the high-risk group presented relatively high distribution levels of monocytes, activated dendritic cells, activated mast cells, and eosinophils but relatively low distribution levels of resting NK cells and resting mast cells. Most of the non-neoplastic population of GBM was composed of infiltrating immune cells, whereas local inflammatory TME promoted tumor aggressiveness and the drug resistance of GBM [25]. For example, some study demonstrated that the frequency of M2 macrophages or microglia in the TME of recurrent GBM increased [26], and dendritic cells presented tumor cell peptides in the GBM, leading to cytotoxic T cells response and secretion of proinflammatory cytokines [25]. NK cells are activated by IL-12 and can kill GBM cells [27]. Moreover, the effectiveness of NK cells in inhibiting systemic metastasis of GBM in xenograft mouse models was reported [28]. Hence, TME immunocyte infiltration under different risk scores had different features and different tumor immune-reactions. The infiltrating immunocytes were closely related with the growth, invasion, and metastasis of GBM.

To elaborate the potential molecular mechanisms of five prognosis PRGs, a ceRNA network was built by searching the database and overlapping prediction results, which was used in recognizing the lncRNA GABPB1-AS1/hsa-miR-519c-5p/CASP3/TP63 regulatory axis. LncRNA is a type of noncoding RNAs with a length of more than 200 nucleotides [29]. Changes in lncRNA expression and its mutation can promote the occurrence and metastasis of tumors [30]. lncRNA plays a key role in TME intracellular signal transduction [31]. GABPB1-AS1 is an lncRNA in the cytoplasm. Recently, many studies have pointed out that lncRNA is a poor prognosis marker of glioma [32], cervical cancer [33], breast cancer [34], and prostate cancer [35]. According to this study, significant differences in GABPB1-AS1 expression was found between tumor and normal tissues. Similarly, Li et al. [36] found high expression of GABPB1-AS1 in the glioma tissues, and in vitro and in vivo experiments have demonstrated that GABPB1-AS1 knockdown reduced the proliferation and invasiveness of glioma cells. According to our results, Hsa-miR-519c-5p can be regulated by GABPB1-AS1 specifically, thus regulating the transcriptional levels of CASP3 and TP63. On this basis, Hsa-miR-519c-5p can control ECM-related signaling pathways (e.g., ECM receptor interaction, adherens junction, and TGFβ signaling pathways) and immune-related signaling pathways (e.g., Toll-like receptor and NOD-like receptor signaling pathways). This demonstrated that ECM and immune inflammation reactions played the key role in occurrence, invasion and metastasis of GBM. The TGF-β in the tumor tissues can inhibit immune cells, decrease local inflammation, and promote the reconstruction of ECM [37]. ECM components and high expression levels on the corresponding cell surface receptors are closely related with the progression of GBM [38]. Toll-like receptors (TLRs) can recognize non-self-molecules and activate the inflammation process. TLR expression had been observed in various samples, such as GBM cases and GBM cell lines, indicating that TLR plays an important role in tumor invasion and metastasis [39]. Therefore, we speculated that the mechanism by which GABPB1-AS1 regulates the transcriptional levels of CASP3 and TP63 and relevant signaling pathways through Hsa-miR-519c-5p might play a crucial role in the occurrence and development of GBM.

## Conclusions

In conclusion, pyroptosis and TME-related independent prognostic markers were screened, and a prognosis risk score formula was established for the first time on the basis of prognosis PRGs. TME immunocyte infiltration characterization and immune response were assessed with ssGSEA, CIBERSORT algorithm, TIMER, and TISIDB database. A ceRNA network was then constructed. This study not only laid the foundation for the further exploration of pyroptosis and TME for the improvement of GBM prognosis but also provided novel insights for clinical immunotherapy and development of novel immunotherapeutic drugs. Deep experimental and clinical studies are still needed to elucidate the molecular mechanism of GBM and clinical applications of biomarkers and immunotherapy.

## Supplementary Information

**Additional file 1: Table S1.** Correlation analysis of prognostic PRGs expression with immunocyte levels in TIMER database.

## Data Availability

The datasets generated and/or analysed during the current study are available in the TCGA (https://portal.gdc.cancer.gov/), GTEx (https://www.gtexportal.org/), GSEA-MSigDB (http://www.gsea-msigdb.org/gsea/login.jsp), Reactome (https://reactome.org/), Harmonizome (https://maayanlab.cloud/Harmonizome/), R software (version 4.1.1, https://www.r-project.org), STRING (version 11.5, https://string-db.org/), Cytoscape software (version 3.8.2, https://cytoscape.org), Gene Ontology (GO) http://geneontology.org/), KEGG (https://www.kegg.jp/kegg/kegg1.html), TIMER (http://timer.cistrome.org/), TISIDB (http://cis.hku.hk/TISIDB), Starbase (http://starbase.sysu.edu.cn/), MirTarbase (http://mirtarbase.cuhk.edu.cn/), Oncolnc (http://www.oncolnc.org/), Lncbase (https://carolina.imis.athena-innovation.gr/diana_tools/web/index.php?r=lncbasev2/) and GEPIA2 (http://gepia2.cancer-pku.cn/).
