# Supplementary material for: Pyroptosis-related prognosis model, immunocyte infiltration characterization, and competing endogenous RNA network of glioblastoma
Source: BMC Cancer. 2022 Jun 3;22:611. doi: 10.1186/s12885-022-09706-x (PMC9166343; doi:10.1186/s12885-022-09706-x)
Supplement: Supplementary file 1 — Additional file 1: Table S1. Correlation analysis of prognostic PRGs expression with immunocyte levels in TIMER database. [file 12885_2022_9706_MOESM1_ESM.docx]

**Table S1.** Correlation analysis of prognostic PRGs expression with immunocyte levels in TIMER database.

| **Gene** | **Immunocytes** | **Cor-value** | **Partical.cor-value** | **P-value** |
| --- | --- | --- | --- | --- |
| CASP3 | Purity | 0.149 | - | 2.23E-03 |
|  | B Cell | - | 0.081 | 1.00E-01 |
|  | CD8+ T Cell | - | 0.109 | 2.65E-02 |
|  | CD4+ T Cell | - | -0.05 | 3.05E-01 |
|  | Macrophage | - | 0.084 | 8.72E-02 |
|  | Neutrophil | - | 0.155 | 1.50E-03 |
|  | Dendritic Cell | - | 0.193 | 6.89E-05 |
| NLRP2 | Purity | -0.212 | - | 1.24E-05 |
|  | B Cell | - | -0.035 | 4.78E-01 |
|  | CD8+ T Cell | - | -0.197 | 4.96E-05 |
|  | CD4+ T Cell | - | -0.11 | 2.40E-02 |
|  | Macrophage | - | -0.099 | 4.31E-02 |
|  | Neutrophil | - | -0.089 | 6.94E-02 |
|  | Dendritic Cell | - | 0.052 | 2.92E-01 |
| TP63 | Purity | -0.097 | - | 4.63E-02 |
|  | B Cell | - | -0.047 | 3.42E-01 |
|  | CD8+ T Cell | - | -0.064 | 1.90E-01 |
|  | CD4+ T Cell | - | -0.143 | 3.34E-03 |
|  | Macrophage | - | -0.167 | 5.93E-04 |
|  | Neutrophil | - | -0.196 | 5.65E-05 |
|  | Dendritic Cell | - | -0.063 | 2.02E-01 |
| GZMB | Purity | -0.211 | - | 1.37E-05 |
|  | B Cell | - | 0.09 | 6.49E-02 |
|  | CD8+ T Cell | - | -0.203 | 3.00E-05 |
|  | CD4+ T Cell | - | -0.174 | 3.50E-04 |
|  | Macrophage | - | -0.007 | 8.94E-01 |
|  | Neutrophil | - | -0.083 | 9.05E-02 |
|  | Dendritic Cell | - | 0.12 | 1.38E-02 |
| CASP9 | Purity | 0.167 | - | 6.00E-04 |
|  | B Cell | - | 0.032 | 5.14E-01 |
|  | CD8+ T Cell | - | -0.029 | 5.51E-01 |
|  | CD4+ T Cell | - | 0.199 | 4.22E-05 |
|  | Macrophage | - | 0.174 | 3.48E-04 |
|  | Neutrophil | - | 0.315 | 4.54E-11 |
|  | Dendritic Cell | - | -0.001 | 9.83E-01 |
